# Development of an Affordable, Sustainable and Efficacious Plant-Based Immunomodulatory Food Ingredient Based on Bell Pepper or Carrot RG-I Pectic Polysaccharides

**DOI:** 10.3390/nu13030963

**Published:** 2021-03-16

**Authors:** Sue McKay, Paul Oranje, Jari Helin, Jean H. Koek, Ellen Kreijveld, Pieter van den Abbeele, Ute Pohl, Gordana Bothe, Maria Tzoumaki, Marcela Aparicio-Vergara, Annick Mercenier, Henk Schols, Ruud Albers

**Affiliations:** 1 Suze Consulting, Voorweg 65, 3233 SJ Oostvoorne, The Netherlands; sue.mckay@xs4all.nl; 2 IMcoMET BV, Marconistraat 16, 3029 AK Rotterdam, The Netherlands; p.oranje@imcomet.com; 3 Glykos Finland Oy, Viikinkaari 6, FI-00790 Helsinki, Finland; jari.helin@glykos.fi; 4 Unilever, Foods Innovation Centre, Bronland 14, 6708 WH Wageningen, The Netherlands; jan.koek@unilever.com; 5 Rijk Zwaan, Burgemeester Crezéelaan 40, P.O. Box 40, 2678 KX De Lier, The Netherlands; e.kreijveld@rijkzwaan.nl; 6 ProDigest BV, Technologiepark 82, 9052 Ghent, Belgium; Pieter.VandenAbbeele@prodigest.eu or; 7 Analyze & Realize GmbH, Waldseeweg 6, 13467 Berlin, Germany; upohl@a-r.com (U.P.); gbothe@a-r.com (G.B.); 8 Nutrileads BV, Bronland 12-N, 6708 WH Wageningen, The Netherlands; maria.tzoumaki@nutrileads.com (M.T.); marcela.aparicio@nutrileads.com (M.A.-V.); annick.mercenier@nutrileads.com (A.M.); 9 Laboratory of Food Chemistry, Wageningen University, Bornse Weilanden 9, 6708 WG Wageningen, The Netherlands; henk.schols@wur.nl

**Keywords:** rhamnogalacturonan-I (RG-I), bell pepper, bpRG-I, carrot, cRG-I, innate immune response, microbiota modulation, short chain fatty acids (SCFA), immunity, viral infections

## Abstract

The prevalence of acute respiratory infections and their impact on quality of life underlies the need for efficacious solutions that are safe, sustainable and economically viable. Polysaccharides in several (traditional) plant extracts have been shown to be immunostimulatory, and some studies suggest beneficial effects against respiratory infections. The aim of this study was to (i) identify the active polysaccharide constituents from affordable and renewable crops (bell pepper and carrot) using activity-guided fractionation, (ii) evaluate in vitro effects on innate immune responses (phagocytosis and cytokine secretion), microbiota modulation and production of short chain fatty acids, followed by (iii) the evaluation of effects of a bell pepper extract enriched for the active component in a human proof of concept study. We identified rhamnogalacturonan-I (RG-I) as the nutricophore responsible for the immunostimulatory activity with substantial structural and functional equivalence between bell pepper (bp) and carrot (c). The in vitro studies showed that bpRG-I and cRG-I comprise similar immune- and microbiota modulatory potential and the human study demonstrated that bpRG-I was well tolerated and enhanced innate immune responsiveness in vivo. This is an important step towards testing the efficacy of RG-I from bpRG-I or cRG-I in an infection trial in humans.

## 1. Introduction

Respiratory infections are recognized now, more than ever, to be a major burden on health with far-reaching socio-economic consequences [1,2,3]. The prevalence of acute respiratory infections and their impact on quality of life underlies the need for efficacious solutions that are safe, sustainable, and economically viable, as well as easy for people to integrate into their daily life, e.g., through incorporation in habitual food products.

Traditional medicinal extracts from a wide range of plants including North American (*Panax quinquefolius*) and Asian (*Panax ginseng*) Ginseng and *Hoodia gordonii* have been used for centuries as traditional remedies against (respiratory tract) infections [4,5,6,7]. Their alleged efficacy is mostly based on preclinical in vitro and animal studies. Large randomized controlled trials are rare. A range of trials done with standardized polysaccharide-enriched extracts of ginseng showed that prophylactic intake modulates innate immune responses [8,9,10,11] and reduces incidence, severity and duration of respiratory viral infections [12,13,14,15]. However, ginseng and other exotic plant extracts meet with regulatory challenges, are often expensive and are not derived from renewable sources [4,16]. Several polysaccharide-enriched extracts from more common renewable crop sources have been reported for their ability to modulate the immune system [17,18], and our own studies identified bell pepper (*Capsicum annuum*) and carrot (*Daucus carota*) as promising plant sources containing particularly active polysaccharides while also being affordable and potentially scalable candidate source crops.

Most acute (respiratory) infections are transient, and the initial host response is driven largely by the innate immune system, with pathogen-associated molecular patterns (PAMPs) initiating NF-κB signaling cascades via pattern-recognition receptors (PRR). Respiratory infections have also been shown to modulate the composition of the gut microbiota, while probiotics and gut microbiota have been shown to modulate pulmonary immune responses via microbial associated molecular patterns (MAMPs) or microbial metabolites such as short chain fatty acids (SCFA). This two-way dialogue, known as the gut–lung axis (GLA), has been demonstrated in humans with recruitment of innate immune cells from the gut to the lungs, and increased numbers of recirculating immunologically active cells [12]. It has been hypothesized that the prophylactic/therapeutic effects of polysaccharide-enriched extracts from ginseng and other traditional plants is mediated via two routes (1) recognition of specific polysaccharide domains by PRRs including toll-like receptors (TLRs), scavenger receptor and C-type lectins like Dectin-1 resulting in priming or training of innate immune responsiveness and (2) microbial fermentation of the polysaccharides (PS), resulting in modulation of the intestinal microbiota composition and production of biologically active metabolites including SCFA [17,18,19,20,21,22,23,24].

Detailed insights into the structure–function relationship of the bioactive domains in polysaccharide-enriched extracts are still largely elusive [17,18,24,25]. This is partly due to differences in experimental methodology, the source material, the extraction process and the variation in how extracts are characterized. The molecular weight, presence and type of side chains, monosaccharide composition and degree of methylation and acetylation can all play a role in determining the biological activity of polysaccharides. A wide range of polysaccharide structural domains have shown biological activity in preclinical models, including glucans, mannans, type I or II arabinogalactans (AG-I, AG-II), and type I or II rhamnogalacturonans (RG-I, RG-II) [18,24,26].

In this study, we used activity-guided fractionation to investigate the immunostimulatory activity of polysaccharide-enriched extracts from bell pepper (*Capsicum annuum*) and carrot (*Daucus carota*). *Hoodia gordonii* extracts were used for comparison in some of the initial experiments. Parallel to upscaling and optimizing the extraction process, we used monosaccharide composition, molecular weight determination and protein nuclear magnetic resonance (^1^H-NMR) in concert with in vitro functional assays to characterize the nutricophore. Our results showed that the moderately acidic pectic polysaccharide fractions with high MW from red bell pepper and carrot had similar immune stimulating and microbiota modulating activity in vitro. We identified this activity to be dependent on the RG-I backbone for both red bell pepper and carrot. A human proof of concept study demonstrated that an extract enriched for RG-I domains from bell pepper (bpRG-I) stimulated innate immune responsiveness in vivo and can modulate gut microbiota in vivo. These results provide an important step towards the development of bell pepper or carrot RG-I derived health ingredients that are safe, sustainable and can be incorporated into habitual food products to support immune function and potentially increase resistance to respiratory infections.

## 2. Materials and Methods

### 2.1. Materials

For initial lab-scale extractions, *Hoodia gordonii* (residual powder after methanol extraction of glycosteroids, Phytopharm Plc, UK), bell pepper (Paprika Mild 80–100, Asta St-Felix Reverte, S.A., Spain) and carrot (ex. R. Steinicke GmbH, Germany) were sourced as dry powder. For larger scale extractions, bell pepper powder (Natural Spices, the Netherlands) and food-grade dried carrot pomace (carrot fibre m20, Greenfield, Poland) were used.

### 2.2. Extraction of Polysaccharide Rich Extracts

#### 2.2.1. Lab-Scale Preparation of Bell Pepper and Carrot Polysaccharide Extracts for Activity-Guided Analyses

For lab-scale extraction of polysaccharide-rich fractions, bell pepper and carrot powder were washed twice for 2.5 h with 85% aqueous ethanol at room temperature and once for 1.5 h at 80 °C. The alcohol insoluble residue (AIR) was collected by centrifugation. The polysaccharides (PS) were extracted by boiling the AIR twice for 3 h in water (AIR:water ratio ca 10), and the aqueous phases were combined and lyophilized. This material is referred to as bell pepper extract and carrot extract.

*Macer treatment:* Polysaccharide extract was dissolved in 35 mM sodium acetate pH 5.0, and MACER 8W, a broad-specificity mixture of polysaccharide degrading enzymes (Biocatalysts Ltd., Cardiff, UK) was added. Reactions were allowed to proceed at 37 °C for 24 h and were stopped by boiling for 5 min. Hydrolysis of polysaccharides was confirmed by ^1^H-NMR spectroscopy.

*Saponification of polysaccharide extracts:* To remove methyl esters and acetyl groups, polysaccharide extracts were incubated in 50 mM NaOH at 4 °C overnight. The solution was then neutralized with acetic acid and concentrated in vacuo. Polysaccharides were isolated by gel filtration and the ester removal was verified by ^1^H-NMR spectroscopy.

*Solid-phase C-18 silica cleaning of PS samples:* PS dissolved in water were run through a 100 mg column of Bond-Elut C-18 (Varian Inc., Palo Alto, CA, USA) preconditioned with one column volume of ethanol and two column volumes of water. The column was eluted with water and the sample obtained was lyophilized. To test LPS removal efficiency, columns were loaded with 15 µg *E. coli 0111:B4* LPS (Sigma–Aldrich, Bornem, Belgium). Pre- and post-column samples were tested at dilutions equal to those used for testing PS-extracts.

#### 2.2.2. Industrial Scale Extraction of Bell Pepper RG-I and Carrot RG-I

The extraction method was optimized to enable industrial scale production and obtain further enrichment of pectic RG-I domains from bell pepper (bpRG-I) and carrot (cRG-I). Commonly used food processing methods were applied including aqueous extraction at 85 °C (bell pepper) or at 45 °C in the presence of Pectinex™ Ultra Mash (Novozymes, Bagsværd, Denmark) food-grade pectinolytic enzymes with pectin lyase and polygalacturonase as main activity (carrot); decanting and filtration to remove non-soluble residues; centrifugation to remove fat (decreaming, bell pepper), ultra- and dia-filtration to remove small molecules (<10 kDa), pasteurization and subsequent spray-drying of the RG-I enriched polysaccharide extracts.

### 2.3. Chemical Composition and Structure

#### 2.3.1. Preparation of Subfractions Using Diethylaminoethyl (DEAE) Anion-Exchange Chromatography (Neutral, Moderately Acidic and Highly Acidic Subfractions)

Polysaccharide extracts were dissolved in 20 mM Tris-HCl, pH 7.5. Insoluble matter was removed by centrifugation and the clear supernatant subjected to anion-exchange chromatography on a DEAE-sepharose column (50 × 150 mm; GE Healthcare, Chicago, IL, USA) equilibrated with 20 mM Tris-HCl, pH 7.5. The column was run isocratically for 30 min, followed by a gradient of 0–1 M NaCl over 30 min, and 1 M NaCl for an additional 40 min. Absorbance at 214 nm was recorded and 25 mL fractions were collected.

#### 2.3.2. Determination of Molecular Weight (MW) by Superdex 200 Size Exclusion Chromatography (HP-SEC)

Polysaccharide samples were dissolved in 0.1 M ammonium bicarbonate and separated by gel filtration chromatography on a Superdex 200 column (either 1 × 30 cm or 5 × 95 cm; GE Healthcare, Amersham, UK). Eluting components were detected by absorption at 214 nm. The polysaccharides were typically isolated into three fractions: MW > 110 kDa, 70–110 kDa and 40–70 kDa. The pooling limits were determined by comparing to elution positions of Dextran standards of 40, 70 and 110 kDa.

#### 2.3.3. Monosaccharide Composition Analysis

The monosaccharide composition of extracts was determined using 2 M aqueous trifluoroacetic acid at 120 °C for 2 h, neutralized with NaOH and subsequently analyzed by high performance anion-exchange chromatography (HPAEC) using pulsed-amperometric detection [27]. To ensure complete hydrolysis, the bpRG-I and cRG-I samples were later also analyzed using methanolysis to release monomers [27].

Uronic acids (UA) in the samples were analyzed after pre-hydrolysis with 72% (*w*/*w*) sulphuric acid at 30 °C for 1 h, followed by hydrolysis with 1 M sulphuric acid at 100 °C for 3 h. The uronic acids released were quantified by using the colorimetric m-hydroxydiphenyl assay automated on an autoanalyzer (Skalar, Breda, The Netherlands) as described before [28].

#### 2.3.4. ^1^H-NMR

For NMR analysis, dry polysaccharide samples were dissolved in deuterium oxide (D_2_O). Spectra were collected on a Varian Unity 500 NMR spectrometer at 296 K and referenced to an internal acetone standard (2.225 ppm). The polysaccharide substitution level and the molar ratios of the different monosaccharide units were analyzed by using the NMR data as described below.

*Rhamnogalacturonan substitution level*: The ratio between 4-substituted rhamnose units and non-substituted units was estimated by integrating the splitted rhamnose –CH_3_ signals. The –CH_3_ protons in 4-substituted rhamnose units resonate around 1.32 ppm, while those of the non-substituted rhamnose units at 1.25 ppm [29].

*Molar ratio of galactans and arabinans*: The molar amount of galactans and arabinans was analyzed by integrating the observed β1,4-galactan H-1 signal (4.64 ppm) and the arabinan H-1 signals (–5Ara H-1, 5.09 ppm; –3,5Ara H-1 5.12 ppm; terminal Araα1-3 5.15 ppm; terminal Araα1-2 5.18 ppm; -2,3,5Ara H-1 5.26 ppm. These integration values were compared to the integrated rhamnose –CH_3_ signals.

*Acetylation level*: The galacturonic acid O-acetyl group –CH_3_ signals reside around 2.07–2.18 ppm, and are easily integrated as typically no interfering signals are present in this area [30]. The O-acetylation level was normalized to the amount of galacturonic acid with 100% being 1 acetyl group per galacturonic acid residue.

*Methyl esterification*: The galacturonic acid methyl esterification level is estimated from the integrated galacturonic acid H-4 signals. The H-4 signal of methyl esterified galacturonic acid unit resides at 4.47 ppm, while the H-4 signal of non-esterified galacturonic acid is at 4.42 ppm.

*Homogalacturonic acid/rhamnogalacturonan-I (RG-I) ratio*: The total amount of galacturonic acid H-4 signals is integrated between 4.42 and 4.47 ppm, and the amount of rhamnose is obtained by integration of the –CH_3_ signals (1.25–1.32 ppm). The H-4 signal of the RG-I specific GalAα1-2 unit is located in the same 4.42–4.47 signal, and its portion has to be deducted from the total H-4 signal. This value is the same as the amount of rhamnose because RG-I is a 1:1 polymer (-4GalAα1-2Rhaα1-4). The remaining H-4 signal represents the share of homogalacturonic acid type GalAα1-4 H-4 signal.

### 2.4. In Vitro Biological Function Assays

#### 2.4.1. Immune Function

##### Phagocytosis Activity

Phagocytosis activity was analyzed with the Phagotest™ Kit (Glycotope Biotechnology, Heidelberg, Germany). Briefly, heparinized human whole blood was incubated with bell pepper or carrot polysaccharide subfractions in polypropylene, U-bottom 96-well plates (Nunc, Roskilde, Denmark) at 37 °C for 45 min. Next, opsonized fluorescein isothiocyanate (FITC)-labelled *E. coli*, diluted in endotoxin free water (Lonza, Verviers, Belgium) was added in a 25:1 ratio to leukocytes and incubated for 6.5 min at 37 °C. Activity was stopped by cooling, external fluorescence was quenched with quencher solution and cells were lysed and fixed. After washing and resuspending the cells in buffer with propidium iodide, samples were measured using a Cytomics FC500 flow cytometer and analyzed using CXP analysis software (Beckman Coulter, Woerden, The Netherlands). Phosphate buffered saline (PBS, Lonza, Verviers, Belgium) and lipopolysaccharide (LPS, 100 ng/mL *E. coli* 0111:B4, Sigma–Aldrich, Bornem, Belgium) were used as negative and positive controls, respectively. Modulation of phagocytosis by polysaccharide samples was calculated as the normalized percentage of FITC-positive granulocytes relative to the dynamic range between constitutive phagocytosis (incubation with PBS, 0%) and the positive control (incubation with LPS, 100%).

##### Cytokine Secretion in Peripheral Blood Mononuclear Cells (PBMC) and Whole Blood from Healthy Donors

*PBMC*: PBMC were isolated from buffy coats of blood using Ficoll-plaque (Amersham, Little Chalfont, UK). PBMC (2 × 10^6^ cells/mL) were incubated in RPMI medium (Gibco™ RPMI 1640 Medium, Waltham, MA, USA) with 0.3 g of RG-I polysaccharides for 20 h (5% CO_2_, 37 °C). Subsequently, supernatants were harvested and cytokines were measured using a bead array (CBA human inflammation kit, BD-Bioscience, The Netherlands) on a flow cytometer (BD FACSCANTO II) according to the manufacturer’s instructions. RPMI was used as negative control and 1 µg/mL LPS (from *E. coli O55:B5*— Sigma–Aldrich, Bornem, Belgium) as a reference to which the results were normalized with LPS as 100%. Data are expressed as % normalized to the response induced by LPS, averaged over 3 different donors for 4 different cytokines.

*Whole blood*: Heparinized whole blood from healthy donors was diluted in cell culture medium and distributed into 96-well micro-culture plates (Greiner, Pleidelsheim, Germany), to which bpRG-I and cRG-I had been added. Commercially obtained apple pectin comprising mainly homogalacturonan and β-glucan were included for comparison. A mixture of LPS (Calbiochem, San Diego, CA, USA) and *Staphylococcus aureus* enterotoxin B (SEB; Bernhard-Nocht-institut, Hamburg, Germany) served as positive control for monocyte and T-cell activation. All experiments were performed in quadruplicate. Supernatants were harvested after incubating the whole blood cultures at 37 °C in a humidified atmosphere containing 5% CO_2_ for 48 h. Replicates were pooled and thoroughly mixed, then aliquots were frozen at −20 °C until analysis. Cytokine testing was performed with Luminex^®^ assays using HumanCytokine MAP A and B kits (Myriad RBM, Austin, TX, USA) according to the manufacturer’s instructions. Graphs show results at 500 μg/mL, values below 0.01 are projected at 0.01 (pg or ng/mL) to enable graphic representation.

#### 2.4.2. Microbiota Modulation

##### Colonic Batch Incubations

Short-term colonic incubations were performed as described in [31]. Briefly, fresh fecal material from a healthy human donor (f, 26y) was collected and after preparation of an anaerobic fecal slurry inoculated at 10 vol% in a sugar-depleted nutritional medium containing 5.2 g/L K_2_HPO_4_, 16.3 g/L KH_2_PO_4_, 2.0 g/L NaHCO_3_ (Chem-lab NV, Zedelgem, Belgium), 2.0 g/L yeast extract, 2.0 g/L pepton (Oxoid, Aalst, Belgium), 1.0 g/L mucin (Carl Roth, Karlsruhe, Germany), 0.5 g/L L-cystein and 2.0 mL/L Tween80 (Sigma–Aldrich, Bornem, Belgium). Test products (bpRG-I and cRG-I) were dosed at 5 g/L and reactors were anaerobically incubated at 37 °C for 48 h. The base medium with no addition of test product and inoculated by the fecal slurry was used as the negative control (blank). All experiments were performed in technical triplicate.

##### Microbial Metabolic Activity

Samples were collected at 0, 6, 24, and 48 h after inoculation from each reactor and pH measurements were performed with a Senseline pH meter F410 (ProSense, Oosterhout, The Netherlands). Short chain fatty acids (SCFA) and branched chain fatty acids (BCFA) were measured by gas chromatography as described previously [32].

##### Microbial Community Composition

Samples were collected at the beginning and at the end of the 48 h of incubation, i.e., for the negative control designated by inoculum (0 h) and base medium (48 h), respectively. Analysis of the microbial community composition was performed by 16S-rRNA targeted Illumina sequencing (LGC genomics GmbH, Berlin, Germany) as described in [33] using primers spanning the V3-V4 hypervariable regions of the 16S rDNA. Results are expressed as proportional abundances (%) at different phylogenetic levels (phylum and family).

### 2.5. Human Randomized, Double-Blind, Placebo-Controlled Proof of Concept Study

#### 2.5.1. Participants and Study Design

Sixty healthy volunteers participated in a randomized, double blind, placebo-controlled proof of concept study carried out in accordance with the Declaration of Helsinki as well as the ICH-GCP guidelines and EU recommendations (CPMP/ICH/135/95). Informed consent was obtained from all participants, ethical approval was granted by “Ethikkommission der Charité” of Berlin, Germany and the trial was registered in the German trial registry as DRKS00010568. The participants were Caucasian males and females aged 20–65 years with body mass index (BMI) of 18.5–29.9 kg/m^2^. Participants were excluded if they had health conditions, used medication or supplements, followed dietary restrictions, or had lifestyle habits that may have interfered with the study (Table S4).

Participants were stratified according to age (<55 and ≥55 years) and gender, and randomization was performed to assign subjects to either the placebo (maltodextrin, Maldex170, TSS Belgium NV, Aalst, Belgium), or bpRG-I groups 0.3 g/day or 1.5 g/day, each mixed with instant cappuccino powder resulting in indistinguishable test articles. Participants were instructed to mix one sachet with a cup of hot water (approximately 100 mL) once per day with the first meal (preferably with breakfast and at the same time of the day) for the duration of the study. Blood samples were collected at baseline (day 0) and on day 5 and 28 (±3) of the study for immediate determination of phagocytosis activity and natural killer cell activity. Stool samples were collected at baseline (day 0) and on day 28 (±3) and stored at −80 °C until microbial community composition was analyzed for the groups receiving 0 or 1.5 g/day of bpRG-I.

Safety and tolerability outcomes were determined at baseline (day 0) and on day 28 (±3 days). Clinical chemistry and hematology markers comprised hemoglobin, hematocrit, erythrocytes, mean corpuscular volume, mean corpuscular hemoglobin, thrombocytes, leucocytes, lymphocytes, neutrophils, eosinophils, basophils and monocyte levels or values. Clinical chemistry included liver function parameters (aspartate aminotransferase, alanine aminotransferase, gamma glutamyl transpeptidase, alkaline phosphatase, total bilirubin), kidney function parameters (creatinine, urea), proteins (total protein, albumin, uric acid), metabolic parameters (fasting cholesterol, triglycerides, glucose, free fatty acids) and highly sensitive C-reactive protein. Urinalysis included protein, glucose, and pH.

The Short Form 12 (SF-12) health survey questionnaire (to assess physical and mental component summary scores) was filled in on day 0 and 28 (±3) by the subjects and the global evaluation of benefit was assessed (globally scaled evaluation with “very good”, “good”, “moderate” and “poor”) [34].

#### 2.5.2. Immune Function

##### Phagocytosis Activity

The quantitative determination of phagocytosis in heparinized whole blood was determined on day 0, 5 and 28 (±3) by means of the Phagotest™ Kit (Glycotope Biotechnology GmbH, Heidelberg, Germany) according to the manufacturer’s instructions.

##### Natural Killer (NK) Cell Activity

The quantitative determination of NK cell cytotoxic activity using heparinized whole blood was determined on day 0, 5 and 28 (±3) by means of the NKtest™ (Glycotope Biotechnology GmbH, Heidelberg, Germany) according to the manufacturer’s instructions. The percentage of target cells (T) killed by effector NK cells (E) with and without prior incubation with IL-2 were determined for different E:T ratios (12.5:1, 25:1, 50:1).

### 2.6. Statistics

For in vitro experiments: data are presented as mean +/− SEM of at least 3 independent experiments. For microbial composition at family level: statistical differences within each family versus the base medium incubation were calculated with a 2-sided student *t*-test, using GraphPad Prism 9.0.1. Differences were found to be significant if *p* < 0.05.

For the proof of concept study: there were no data available for a valid sample-size estimation so a sample size of 20 people per treatment (1:1:1) was chosen for feasibility reasons. For continuous endpoints, exploratory two-sided 95%-confidence intervals and two-sided *p*-values for within and between-group treatment differences were calculated for the absolute change from baseline, using an ANCOVA model with the fixed effect factor for the treatment group and with the baseline value as covariate. In addition, exploratory two-sided *p*-values for within- and between-group treatment differences were calculated with the nonparametric Wilcoxon signed-rank test and the Wilcoxon rank-sum test by analyzing the rank sums. For global evaluation, the non-parametric Fisher’s exact test was used for exploratory comparison of treatment groups. Statistical programming and analyses were performed using the statistical software system SAS^®^.

## 3. Results

### 3.1. Polysaccharide Extracts Stimulate Immune Function In Vitro

**Figure 1:**
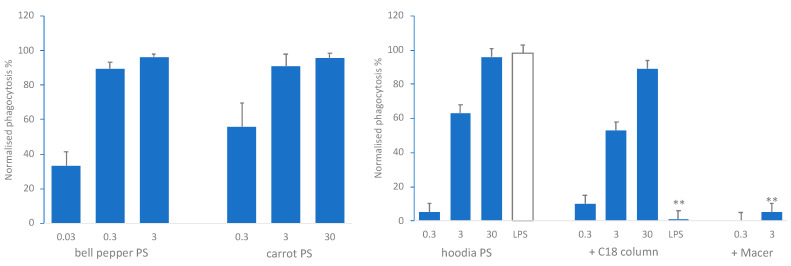
Left: stimulation by bell pepper-PS and carrot-PS of in vitro phagocytosis of fluorescently labelled *E. coli* by phagocytic cells from human blood. Right: stimulation by hoodia PS extracts (and 50 ng/mL LPS as control) before and after passage over C18 column. +Macer indicates effect of hoodia-PS after 24 h incubation with Macer-8W enzyme mix. Results are expressed as % of stimulation by positive control (average + SEM of three donors). LPS +C18 column is significantly different from LPS, and 3 µg/mL hoodia PS + Macer from the same concentration without Macer, (** *p* < 0.01).

Hot water extracts from bell pepper, carrot and hoodia enriched for polysaccharides (PS) dose dependently stimulated phagocytic activity by phagocytic cells (mainly neutrophils) from human blood in vitro (Figure 1). Pretreatment of the hoodia-PS samples with a C18 column to remove hydrophobic compounds did not change this effect, whereas the LPS stimulating effect on phagocytosis was abolished showing that LPS was effectively removed by this pretreatment. Incubation of hoodia-PS with a range of polysaccharide hydrolyzing enzymes (Macer-8W) effectively hydrolyzed the polysaccharides (not shown) and abolished the stimulating effect on phagocytosis (Figure 1, right). These results clearly indicate that the effect on phagocytosis was due to the PS in the extract and not due to (microbial) contaminants, which was consistent with the reproducible nature of the effects over numerous experiments. Comparable dose-dependent in vitro stimulation of innate immune responsiveness was also observed for bell pepper and carrot extracts when assessing phagocytic activity with the human leukemia HL60 phagocytic cell line and natural killer (NK) cell activity using human PBMC (data not shown). In addition, incubation of PBMC with these PS extracts resulted in expression of activation markers indicating effects on monocytes, dendritic cells (DC) and NK cells, but not T and B lymphocytes (data not shown).

### 3.2. Activity-Guided Identification of the Immunomodulatory Nutricophore from Hoodia, Bell Pepper and Carrot Polysaccharide-Enriched Hot Water Extracts

#### 3.2.1. Characterization of Polysaccharides Extracts by ^1^H-NMR

**Figure 2:**
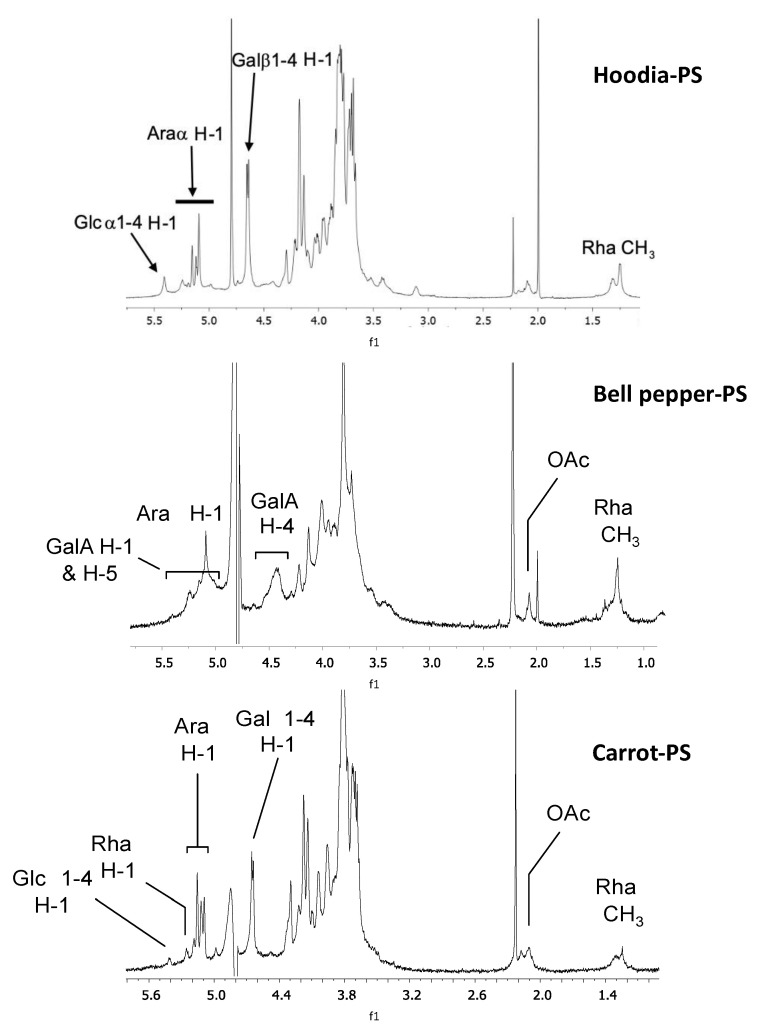
^1^H-NMR spectra of mildly acidic DEAE subfractions of hoodia-PS (top), bell pepper-PS (middle) and carrot-PS (bottom). Signals are assigned to rhamnosyl (Rha), galacturonyl (GalA), galactosyl (Gal), arabinosyl (Ara) and glucosyl (Glc) residues based on external standards.

To identify the immunomodulatory (sub)fraction(s) within the crude PS extracts, hoodia, bell pepper and carrot polysaccharide-enriched extracts were subjected to DEAE anion-exchange chromatography. Three fractions were obtained, representing a neutral fraction eluting with water and two acidic (i.e., negatively charged) fractions being eluted with increasing NaCl concentration. The highest phagocytosis stimulating activity was consistently observed in the mildly acidic sub-fractions. The ^1^H-NMR spectra of these sub-fractions for hoodia-PS, bell pepper-PS and carrot-PS are shown in Figure 2. These spectra reveal the abundant presence of galacturonic acid (GalA), rhamnose (Rha), arabinose (Ara), and galactose (Gal), monosaccharide constituents typical for pectins [35].

**Figure 3:**
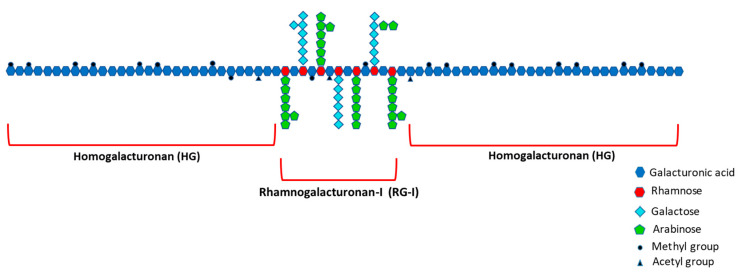
Schematic representation of the main pectin domains to aid interpretation of results below. Pectin molecules are high-molecular-weight polysaccharides consisting of a backbone of two main covalently linked repeating structural units: the smooth homogalacturonan (HG) subunit and the highly branched (‘hairy’) rhamnogalacturonan-I (RG-I) subunit. The HG subunits make up 65% of the pectin molecule and the RG-I subunits constitute approximately 20% to 35% [36,37]. The HG regions consist of α-1,4-linked D-galacturonic acid monomers, whereas the RG-I domains have a backbone of the repeating disaccharide [-α-1,4-D-galacturonic acid-α-1,2-L-rhamnose-] [38]. Depending on the plant species, the rhamnose residues in the RG-I backbone are substituted with β-1,4-D-galactan, branched arabinan α-1,5-linked L-arabinofuranose units with additional L-arabinofuranose side-chains or arabinogalactans [39]. Two minor pectin structural elements xylogalacturonan and rhamnogalacturonan-II are not indicated in this figure.

The structural features of pectic polysaccharides largely determine their functional properties. Figure 3 provides a schematic representation of the main pectin domains homogalacturonan (HG) and RG-I and their monosaccharide constituents. Both the plant source and the extraction method are important factors determining physiochemical properties and bioactivity of pectic polysaccharide-enriched extracts. These factors also influence extraction yield, availability, cost, and ability to produce from sustainably sourced material at large scale [18,24].

#### 3.2.2. Effect of Charge and Molecular Size on In Vitro Phagocytosis Activity

The DEAE sub-fractions of hoodia-PS, bell pepper-PS and carrot-PS were subjected to Superdex 200 size exclusion chromatography (HP-SEC) to further separate the fractions based on molecular weight (MW).

The strongest effect on in vitro phagocytosis activity was consistently found in mildly acidic, higher MW fractions (>110 kDa and 70–110 kDa). Table 1 summarizes sugar composition of the size separated subfractions of selected mildly acidic material as deduced from the ^1^H-NMR spectra and an indication of their potency to stimulate phagocytosis. All sub-fractions contained signals for rhamnose in combination with arabinose and galactose except bell pepper-PS where the galactosyl residues could not clearly be identified due to overlapping signals. The sugar composition obtained suggests the presence of RG-I structures substituted with arabinan and, in case of hoodia and carrot, also galactan as side chains [40]. The least active lower MW fractions (Table 1) contained the most, partially methylated, GalA residues. In contrast, the arabinan and in hoodia-PS and carrot-PS, galactan signals were lower in these fractions, implying that ‘smooth’ partially methylated HG constitutes the major part of these lower MW fractions, whereas the immunostimulatory higher MW fractions contained more ‘hairy’ RG-I regions decorated with arabinan and for, hoodia and carrot, galactan side chains.

Both high MW RG-I and lower MW HG, domains were found in the same (weakly) acidic fraction upon DEAE chromatography, suggesting that their negative charge density was similar. Indeed, in HG, only some galacturonate carboxylate groups of the repeating units carry a negative charge, the remainder being methyl esterified. In RG-I, the charge due to charged GalA carboxylate groups is ‘diluted’ by alternation with rhamnose, some of these substituted with Gal or Ara containing side chains.

#### 3.2.3. Effect of pH on Extraction of Bell Pepper and Carrot Polysaccharides

Further research focused on bell pepper and carrot pectic polysaccharides as these crops enable affordable, large-scale production of immune stimulating polysaccharides from renewable sources. Since the immunomodulatory activity of bell pepper-PS and carrot-PS appeared to reside in the RG-I domains, PS were extracted under slightly alkaline conditions in bicarbonate (pH 7.5–8) to promote degradation of methyl-esterified homogalacturonan domains through beta-elimination [41]. For bell pepper-PS, yield and MW distribution after hot water or alkaline extraction were comparable (Supplemental Table S1 and [42]). However, carrot-PS extraction in boiling water resulted in gel formation which was not observed during alkaline extraction which resulted in a higher yield (20% vs. 9% in water) and a larger fraction in the higher MW region (33% vs. 9% and 23% vs. 16% for the >110 kDa and 70–110 kDa fractions, respectively). The GalA/Rha ratio was highest in the water extracted carrot-PS, possibly explaining the problem with gelling due to the HG domains. Alkaline extraction indeed resulted in enrichment for RG-I domains (lower GalA/Rha), compared to extraction in hot water, and this effect was most pronounced for carrot-PS (Table 2) concurrent with the strong increase in yield and average MW of extracted material (Supplemental Table S1). Alkaline extraction did not change decoration with galactans (Gal/Rha), but the arabinan side-chains (Ara/Rha), methyl- and acetyl esters were completely removed during alkaline extraction. None of these structural changes affected the phagocytosis stimulating activity of the extracts (Table 2). This is congruent with the finding that treatment of hot water PS extracts with enzymes to selectively remove the (neutral) side chains and saponification with sodium hydroxide to remove acetyl and methyl groups did not impact the stimulatory activity on phagocytosis (data not shown).

Overall, the results indicate that the mildly acidic high MW fraction of bell pepper contained pectic HG stretches and RG-I domains with relatively short arabinan side chains. The high MW fraction of hoodia and carrot showed RG-I with longer side chains consisting of both galactose and arabinose. Varying degrees of O-acetylation and O-methylation were observed. Although further work is required to fully elucidate the structure function relationship, results suggest that the structural element for the stimulation of phagocytosis in hoodia-PS, bell pepper-PS and carrot-PS contains an RG-I core, optionally substituted with glycosyl side chains, methyl esters and acetyl groups.

### 3.3. Monosaccharide Composition Analysis of Bell Pepper and Carrot

#### 3.3.1. Lab-Scale Polysaccharide-Enriched Hot Water Extracts

To further characterize the structural element of RG-I stimulating phagocytosis, monosaccharide compositions of bell pepper-PS and carrot-PS were determined after hydrolysis. The results in Table 3 confirm the presence of monosaccharides typical for pectins with 83% and 68% GalA in bell pepper-PS and carrot-PS, respectively. Both extracts also contain substantial amounts of Rha, Gal and Ara. In the extracts, 15% and 25% of the monosaccharides are present as RG-I domains for bell pepper-PS and carrot-PS, respectively. Monosaccharide composition results confirmed the data from the ^1^H-NMR analyses and indicate that RG-I in bell pepper and carrot both contain relatively short arabinan side chains with an Ara/Rha ratio of approximately 2. Unlike ^1^H-NMR, where galactosyl residues could not be identified due to overlapping signals, monosaccharides analysis indicates that bell pepper-PS contains galactose (ratio Gal/Rha = 2), but substantially less than hot water extracted carrot-PS (ratio Gal/Rha = 6).

#### 3.3.2. Industrial Scale RG-I-Enriched Extracts

As the effect on phagocytosis appeared to be linked to the backbone of pectic RG-I domains, the extraction process was further optimized to further enrich RG-I and was at the same time scaled up to industrial scale unit operations. At the industrial scale, bpRG-I could be readily extracted with hot water from bell pepper, although the sugar composition showed that the extract still contained a relatively high amount of Glc due to incomplete removal of small soluble sugars during ultra-filtration (last step before drying). Importantly, the GalA/Rha ratio dropped from 32 to 19 indicating enrichment of RG-I domains without major changes in Ara/Rha and Gal/Rha ratios (Table 4) compared to the lab-scale extractions (Table 3). To avoid pectin gelling and to increase the yield during production of cRG-I, hydrolysis was catalyzed with a food-grade pectic enzyme preparation. The resulting cRG-I was strongly enriched in RG-I domains (74%) with a GalA/Rha ratio of 2 demonstrating that most HG was removed. The enzymes also ‘shaved’ (partially hydrolyzed) the Gal side chains resulting in Gal/Rha and Ara/Rha ratios very similar to those in bell pepper.

### 3.4. Functional/Biological Activity of bpRG-I and cRG-I In Vitro

#### 3.4.1. In Vitro Cytokine Secretion—PBMC and Whole Blood Assay

Extracts of pectic polysaccharides from various plants have been shown to activate the immune system by initiating cellular responses such as phagocytosis and natural killer cell activity and triggering molecular events such as secretion of cytokines and reactive oxygen species [24,43,44]. To compare their in vitro immunomodulatory potential, bpRG-I and cRG-I were added to cultures of isolated human peripheral mononuclear cells (PBMC) and their impact on the secretion of cytokines into the culture medium was measured.

**Figure 4:**
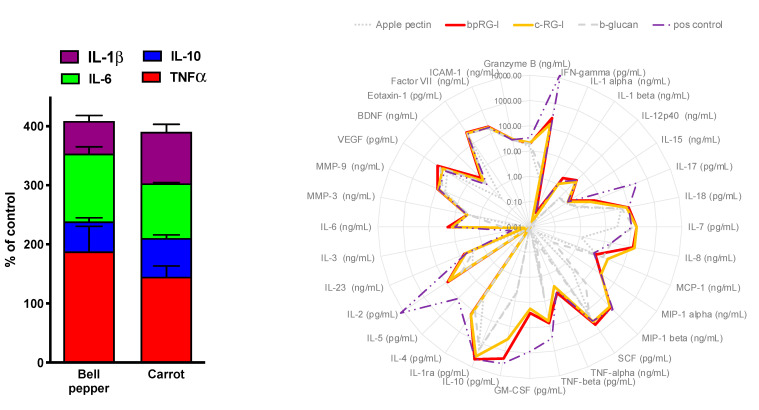
Left: bpRG-I and cRG-I (300 μg/mL) induced comparable cytokine secretion profiles in PBMCs. Average from three healthy donors normalized to LPS standard in those donors. Right: Secretome profiles in whole blood induced by 500 μg/mL bpRG-I (red) and cRG-I (orange), apple pectin (mainly HG, dotted), β-glucan (dashed), and positive control (LPS + SAB; dash, dot dot). Values below 0.01 pg or ng/mL are projected at 0.01 to enable graphic representation.

Figure 4 (left) shows that bpRG-I and cRG-I stimulated comparable levels of TNFα, IL-10, IL-6 and IL1β secretion, which was dose-dependent (not shown). More in-depth comparison in a whole blood assay indicated that, over a wide range of cytokines and chemokines, bpRG-I and cRG-I induced very similar secretome profiles, which are clearly distinct from those induced by apple pectin (>65% homo galacturonan), which had very low activity, β-glucan, and LPS/SEB (positive control; Figure 4, right).

Both bpRG-I and cRG-I (strongly) increased the secretion of an IFNγ, IL10, IL4 (stimulation index over negative control, SI > 500), IL7, IL8, MIP-1α, MIP-1β, TNFβ, GM-CSF, IL2 and IL6 (SI: 100–500), IL1β, IL17, MCP-1, TNFα, BCNF (SI: 10–100) and IL12-p40 (SI: 5–10) (Supplemental Table S2). This profile predominantly shows mediators linked to innate immune responsiveness and comprises factors with pro- as well as anti-inflammatory properties. The similar secretome profile (Figure 4 right) induced by bpRG-I and cRG-I in this complex organotypic in vitro system involving all cells present in human blood suggests that bpRG-I and cRG-I are very similar in their potential to modulate immune function.

#### 3.4.2. Effect of bpRG-I and cRG-I on Gut Microbiota In Vitro

**Figure 5:**
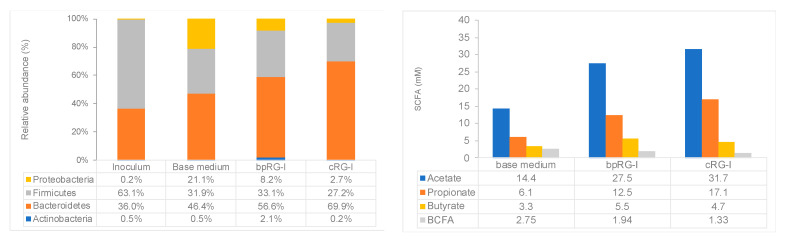
**Left**, effect on microbiota community composition at phylum level (expressed as relative abundance). **Right**, levels of branched-chain fatty acids (BCFA) resulting from protein fermentation were decreased in cultures containing bpRG-I or cRG-I. Globally, the results of these batch cultures show that bpRG-I and cRG-I are effectively fermented resulting in similar profiles of SCFA with cRG-I inducing a slightly stronger effect.

The complex chemical structure of RG-I is not degraded by host digestive enzymes but is known to be fermented by the gut microbiota [45]. To compare their fermentation profiles, bpRG-I and cRG-I were added to the base medium in batch cultures for 48 h inoculated with human fecal microbiota. Both extracts were fermented rapidly as pH decreased already over the first 6 h (pH of 6.18, 6.15 and 6.49 for bpRG-I, cRG-I and control (base medium, no RG-I added), respectively) to reach a final value of 6.04 for bpRG-I and 5.84 for cRG-I compared to 6.41 for the control at 48 h. The microbiota of the fecal inoculum mainly consisted of Bacteroidetes and Firmicutes and became enriched in Proteobacteria by the end of the incubation (±21%) (Figure 5, left panel, base medium). In contrast, bpRG-I and cRG-I extracts induced a shift in microbiota composition over 48 h leading to an increase in the proportional abundance of Bacteroidetes and concomitant decrease in Firmicutes and Proteobacteria as compared to the base medium (Figure 5 left, phylum level). At the family level, this was mainly driven by elevated relative levels of *Bacteroidaceae,* and reduced levels of *Lachnospiraceae* and *Enterobacteriaceae* (Supplemental Table S3).

### 3.5. Immunomodulatory Activity and Safety of bpRG-I in Human Proof of a Concept Study

Prior studies have shown that standardized PS-enriched extracts from ginseng enhance innate immune responsiveness measured as phagocytosis and NK cell activity in humans [8,10,46]. In vitro experiments have shown that RG-I domains are among the main immunomodulatory PS in ginseng extracts [46]. We identified that RG-I from bell pepper and carrot enhances phagocytosis and NK cell activity in vitro, similar to RG-I from hoodia and ginseng. To test if RG-I domains derived from a safe, sustainable low-cost plant source can be used as food ingredient to enhance innate immune responsiveness in vivo, a randomized, placebo-controlled proof-of-concept study was conducted to evaluate the dose effect of bpRG-I on tolerability, phagocytosis and NK cell activity in humans.

#### 3.5.1. Study Subjects

Sixty healthy adults (20–65 years) meeting inclusion and exclusion criteria (Supplemental Table S4) were randomly assigned to receive placebo, low dose (0.3 g/d) or high dose (1.5 g/d) bpRG-I. Following inclusion, mild dietary restrictions on supplements and food products with possible immune modulating effects applied. Subjects consumed placebo (maltodextrin) or bpRG-I supplement premixed with instant cappuccino powder taken daily in hot water with breakfast for 28 (±3) days. The dose of 0.3 g/d bpRG-I was based on the 0.2 to 0.4 g/d ginseng polysaccharides used in earlier studies [10,13,15,47,48] and 1.5 g/d was arbitrarily selected to be five times higher.

**Figure 6:**
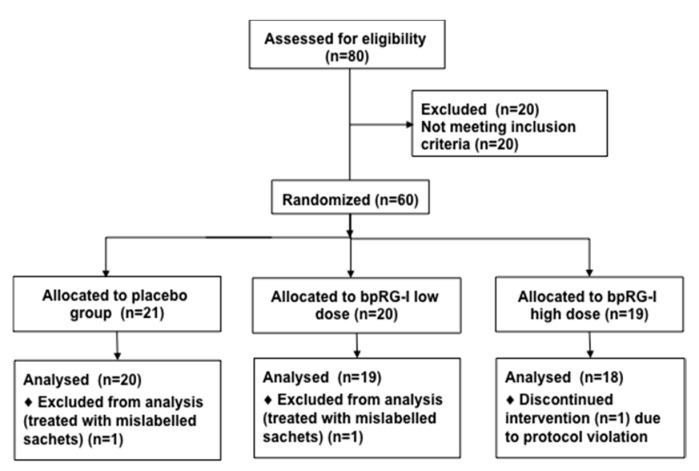
Disposition of subjects.

One subject prematurely discontinued the study due to protocol violation (intake of 3 sachets daily instead of one) and two subjects received mislabeled sachets and were therefore excluded during blind review. Post hoc verification confirmed that only these two subjects received mislabeled sachets. The remaining 57 subjects were included in the analysis (Figure 6). Average age at baseline was 49 ± 12 years and 68% of participants were female. Baseline demographic and anthropometric data were not different between groups (Supplemental Table S5).

#### 3.5.2. Safety and Tolerability

Overall, bpRG-I was very well tolerated as determined by the global assessment of tolerability, with both the subject and the investigator rating “very good” for all subjects except one subject with a “moderate tolerability” at the low dose. Seven treatment emergent adverse events (TEAEs) were reported in four subjects with three in the placebo group (1 of mild, moderate and severe intensity each), 4 of mild intensity in the low dose group and none in the high dose group. The only TEAE considered ‘possibly related’ to treatment was one case of mild flatulence in the low dose group. Hematology, clinical chemistry and urinalysis revealed similar or even less frequently minor abnormalities (values out of normal reference range) after both bpRG-I doses compared to placebo. In addition, no clinically relevant changes were observed in blood pressure and pulse rate. These results indicate that bpRG-I at both doses was well tolerated and is safe for human consumption. The SF-12 physical and mental component summary scores showed no relevant changes within or between treatment groups. The global evaluation of benefit was rated as very good (39/57 subjects) or good (18/57 subjects) with no differences between treatment groups.

#### 3.5.3. Effect of bpRG-I on Innate Immune Responsiveness and Fecal Microbiota Composition

**Figure 7:**
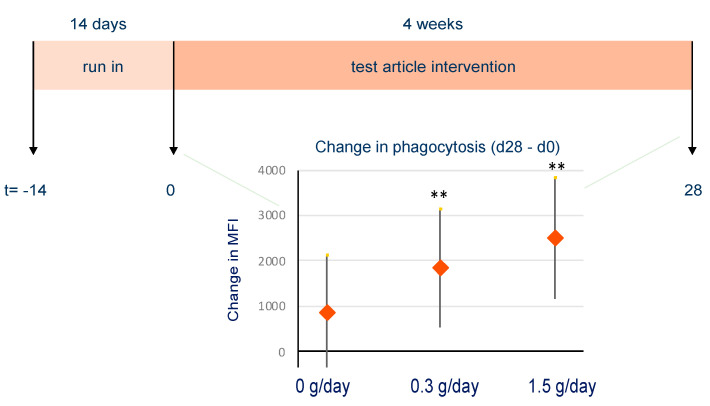
Phagocytic activity of granulocytes as the change on day 28 versus baseline of the median fluorescent intensity. ** *p* < 0.01.

To assess the effect of the intervention on phagocytic activity, the percentage of phagocytic cells involved in phagocytosis (% of cells with internalized fluorescently labelled *E. coli*) and the level of phagocytic activity (mean fluorescent intensity, MFI) were determined. Under the conditions tested, more than 95% of the granulocytes was already engaged in phagocytosis at baseline (day 0). However, on day 28, a slight increase in the percentage of phagocytizing granulocytes in both the placebo (*p* = 0.0492) and high dose bpRG-I group (*p* = 0.0113) was found. The phagocytic activity of granulocytes measured as MFI increased significantly in the low (*p* = 0.0062) and high (*p* = 0.0004) dose bpRG-I groups compared to placebo on day 28, as shown in Figure 7, with a 15% increase in the low dose and 20% increase in the high dose group. These effects were not yet apparent on day 5. The phagocytic activity of the much less abundant and more difficult to identify monocytes showed considerable day-to-day variability at all time points (data not shown).

The significant bpRG-I dose-dependent increase of phagocytosis by granulocytes after four weeks (28 days) is in line with data from trials with ginseng-PS in which similar enhancement of phagocytic activity was observed [8,47].

NK cell activity was also assessed using ex vivo incubations of fresh PBMC (effector cells) with labelled K562 target cells at different ratios in the absence or presence of added IL-2. Overall, NK cytotoxic activity was highly variable between days making it impossible to detect statistically significant changes (data not shown). Scaglione et al. [8,10] have previously shown that an intervention with ginseng resulted in a marginal increase in NK cell activity after four weeks and more convincingly after eight weeks, while Cho et al. showed an increase in NK activity after 8 and 14 weeks [47], suggesting that the time frame for our intervention may have been too short to detect significant changes in NK activity.

## 4. Discussion

This paper describes a series of experiments starting with activity-guided fractionation of PS enriched extracts from bell pepper and carrot shown to have in vitro immunomodulatory effects. Extracts were separated based on charge and MW, tested for activity using an in vitro assay for phagocytosis, and the most active sub-fractions were analyzed using ^1^H-NMR and monosaccharide composition analysis. Pectic RG-I domains were identified as the biologically active nutricophore responsible for the stimulation of phagocytosis. Extensive enzymatic degradation of all PS abolished the stimulatory activity whereas selective removal of arabinan (lower Ara/Rha) and galactan (lower Gal/Rha) side chains of the RG-I domains, of HG domains (lower GalA/Rha ratio) or removal of methyl esters and acetyl groups did not appreciably reduce the stimulatory effect. This suggests that the structural element for stimulation of phagocytosis in bell pepper-PS and carrot-PS contains an RG-I core comprising alternating α-1,2-L-rhamnose-α-1,4-D-galacturonic acid-units, optionally substituted with arabinose and galactose side chains, methyl esters and acetyl groups. This is in agreement with the finding of Zhang et al. who showed that stimulation of phagocytosis by ginseng-PS was due to the RG-I backbone and was not affected by removal of sidechains [49]. However, secretion of nitric oxide and lymphocyte proliferation was reduced after removal of the Ara and Gal residues [49]. Others have found that, for pectin fragments from various sources and depending on the readout used (residual) HG, side chains or esterification (patterns) may, in part, determine activity [17,23,30,50]. Further studies using targeted modification of purified and properly characterized pectic RG-I domains possibly complemented with synthesis of defined sub domains and testing in rigorously standardized assays are required to elucidate the fine structure–function relationship(s).

Identification of pectic RG-I domains as immunomodulatory constituents of the bell pepper-PS and carrot-PS is in line with the growing evidence that pectin derived PS, especially RG-I domains of a wide variety of plants impact on both the host immune system and gut [18,23,24]. However, until recently, these data were largely limited to in vitro experiments, using exotic plants not abundantly present in the food chain and relying on extraction methods that cannot easily be scaled to sustainable industrial production of RG-I. Therefore, we decided to produce RG-I enriched extracts from bell pepper and carrot using a scalable and sustainable production process. The resulting bpRG-I and cRG-I extracts were strongly enriched for RG-I domains with some residual HG in bpRG-I (GalA/Rha: 10) and little residual HG in enzymatically extracted cRG-I (GalA/Rha: 2). Both RG-I variants appear to similarly contain short arabinose and galactose side chains attached to the rhamnose molecules (Ara/Rha 1.6 and 2.4, Gal/Rha 1.7 and 1.4, for bpRG-I and cRG-I, respectively).

Both bpRG-I and cRG-I displayed immunomodulatory activity inducing very similar cytokine secretion profiles when added to PBMC or whole blood cultures. The cytokine profiles are indicative of effects predominantly on the innate immune system and include mediators such as IL6, TNFα, IL17, IL10, IFNγ and IL12. Interestingly, a recent study by Sun et al. indicates that carrot pomace PS stimulated activation of myeloid dendritic cells leading to increased production of IL6, TNFα, IL17 and IL10 in a mixed lymphocyte reaction [51]. The carrot pomace PS were not characterized in that paper, but the extraction was similar to our method for extraction of cRG-I. When given orally to mice immunosuppressed with cyclophosphamide, carrot pomace PS resulted in improved antibody titers to a flu vaccine and increased recovery of depleted NK cells and dendritic cells. The latter appeared to be phenotypically changed resulting in enhanced IL12 and IFNγ production following ex vivo restimulation [51]. These findings in mice corroborate with our preliminary data indicating that incubation of human PBMC with bell pepper-PS and carrot-PS results in expression of activation markers on monocytes, NK cells and dendritic cells, but not T or B lymphocytes (not shown).

Increasing evidence also suggests potent microbiota modulating properties of pectic polysaccharides including RG-I domains [17,43,44,45,52]. In vitro batch cultures with bpRG-I and cRG-I confirmed that both were fermented by human fecal microbiota and induced a shift in relative microbial community composition, increasing the abundance of Bacteroidetes and concomitantly decreasing the relative levels of Firmicutes and Proteobacteria, and enhanced production of SCFA. Analysis of the changes in fecal microbiota composition over the 4-week period in the proof-of-concept study indicated that bpRG-I also modulated the microbiota composition in the 1.5 g/day group (manuscript in preparation).The multi-faceted roles of SCFA as modulators of immune responsiveness is extensively covered elsewhere [21,53,54]. In brief, SCFA are used by colonocytes as a major energy source and influence gene expression necessary for epithelial barrier and defense functions. Second, through interaction with G-protein-coupled receptors and histone deacetylases, SCFA modulate innate immune cells such as macrophages, neutrophils and DCs, through signaling cascades and epigenetic modification. Third, SCFA also affects antigen-specific adaptive immunity mediated by T-cells and B-cells.

Overall, results from our in vitro experiments indicate that bpRG-I and cRG-I have a similar potential to (1) directly modulate immune responsiveness presumably by recognition via PRR leading to priming or training of innate immune responsiveness and (2) indirectly modulate immune function via SCFA and other potentially bioactive metabolites produced by fermentation of RG-I by the gut microbiota. To explore if these potential effects also occur in vivo in humans after oral ingestion, a proof-of-concept trial was conducted to test the dose-dependent effect of bpRG-I given for four weeks as a dietary supplement. This randomized, double-blind, placebo-controlled trial showed that treatment with bpRG-I was well tolerated at both doses (0.3 g/d and 1.5 g/d) and dose-dependently stimulated ex vivo phagocytic activity by blood granulocytes. In addition, it was found that, despite the low dose, the addition of 1.5 g/day RG-I to the daily diet resulted in shifts in microbial community composition (manuscript in preparation) as was seen in the in vitro fecal fermentation studies.

Collectively, the data show that bpRG-I and cRG-I affect innate immune responsiveness, with effects beyond the local intestinal environment as demonstrated by the stimulation of ex vivo phagocytic activity of PBMC. In cyclophosphamide treated mice, oral consumption of carrot pomace polysaccharides was shown to enhance maturation, expansion, and function of myeloid DC in the spleen, thus increasing the efficacy of an injected influenza vaccination [51]. These findings are congruent with the hypothesis that oral consumption of specific plant polysaccharide domains such as RG-I are recognized by PRR of local myeloid cells surveilling the luminal content, which leads to training of these cells, altering their response phenotype. Whether this represents mere priming or involves the characteristic epigenetic and metabolic changes defining innate training [55] remains to be established. Either way, cells modulated in the gut can recirculate through lymph and blood to other mucosal surfaces such as the respiratory tract mucosae. In addition, it was shown that RG-I domains are fermented by the gut microbes, changing microbiota composition and leading to enhanced production of SCFA and other potentially beneficial metabolites. Such biologically active mediators have important effects on local immune responsiveness [26,45,56], and also act beyond the intestinal tract and thus may modulate immune responsiveness to acute respiratory infections [57,58,59]. To what extent prophylactic use of bpRG-I or cRG-I as dietary supplement augments innate immune responsiveness and contributes to increasing resistance to acute respiratory infections remains to be established in properly designed clinical studies.

Here, we provide a first indication that RG-I enriched PS extracts from sustainable plant sources can enhance innate immune function and modulate the gut microbiota not only in vitro, but also in a human proof of concept study (given as dietary supplement). This provides a strong rationale to perform larger randomized placebo-controlled intervention studies to test if ingestion of RG-I from bell pepper and carrot indeed trains innate immune cells, affecting their responsiveness, modulates microbiota composition and function, and in doing so contributes to increased protection to respiratory infections.

## 5. Conclusions

Both in vitro data and the exploratory proof of concept trial support the biological effect of RG-I derived from an affordable and sustainable food source. RG-I enriched extracts prime innate immune responses and display a dual mode of action by exerting (1) an immunomodulatory effect on phagocytosis, a biomarker previously shown in some studies with ginseng-PS to be associated with protective effects against respiratory infections, and (2) a microbiota modulatory effect, with concomitant enhanced production of SCFA. RG-I from bell pepper and carrot showed similar immune and microbiota modulatory activities. Both bpRG-I and cRG-I appear to be efficacious solutions that are safe, sustainable and economically viable, and could easily be applied as food ingredients and integrated into dietary supplements and food products. These results provide an important step towards testing the efficacy of RG-I from bell pepper or carrot in a fully powered respiratory infection trial in humans.

## Figures and Tables

**Table 1:** Sugar composition relative to Rha signal based on ^1^H-NMR data of mildly acidic size-separated subfractions of hoodia-PS, bell pepper-PS and carrot-PS, and potency to stimulate in vitro phagocytosis.

	^1^H-NMR Signal							Phagocytosis (Concentration)	
Material MW Fraction	Rha 4-OH	GalA ^1^	β1-4Gal ^2^	Ara ^2^	Rha 4-OH subst ^3^	O-Ac ^4^	GalA OCH_3_^5^	3 μg/mL	30 μg/mL
**Hoodia-PS**								**+**	**++**
>110 kD	1	1	5	3	50%	100%	n.d.	++	++
70–110 kD	1	6	6	1	30%	10%	46%	+	++
40–70 kD	1	8	5	2	25%	7%	40%	±	+
**Bell pepper-PS**								**++**	**++**
>110 kD	1	4	n.d.	3	20%	8%	40%	++	++
70–110 kD	1	9	n.d.	2	30%	10%	40%	++	++
40–70 kD	1	21	n.d.	1	25%	4%	40%	-	+
**Carrot-PS**								**+**	**++**
>110 kD	1	1	12	11	55%	150%	n.d.	+	++
70–110 kD	1	3	7	5	50%	35%	45%	-	+
40–70 kD	1	20	3	4	40%	10%	30%	-	-

^1^ As measured from GalA H-4 signal area, ^2^ mol/mol compared to Rha, ^3^ measured from the Rha CH_3_ signal, ^4^ O-acetylation of GalA (1 OAc/GalA set as 100%), ^5^ GalA methyl esterification level as estimated from the GalA H-4 signal splitting, n.d.: not distinguishable. In vitro phagocytosis stimulating activity is expressed as average normalized % phagocytosis of at least 2 blood donors, -: 0–20%, +/-: 20–40%, +: 40–80%, ++: >80% stimulation of positive control (LPS).

**Table 2:** Sugar composition relative to Rha signal based on ^1^H-NMR data of bell pepper-PS and carrot-PS after extraction in hot water or bicarbonate (pH 7.5–8), and potency to enhance in vitro phagocytosis.

		Composition					Phagocytosis	
Material	Extraction	GalA/Rha	Ara/Rha	Gal/Rha	GalA-meth	GalA-acet	3 μg/mL	30 μg/mL
bell pepper-PS	water	6.9	4.4	0.0	50%	20%	++	++
	NaHCO_3_	5.3	0.0	0.0	0%	0%	++	++
carrot-PS	water	10.9	11.8	5.3	60%	20%	++	++
	NaHCO_3_	7.6	0.0	5.3	0%	0%	++	++

Monosaccharide ratios and degree of methylation are determined from the ^1^H-NMR spectra. In vitro phagocytosis stimulating activity is expressed as average normalized % phagocytosis of at least 2 blood donors, -: 0–20%, +/-: 20–40%, +: 40–80%, ++: >80% stimulation of positive control (LPS).

**Table 3:** Monosaccharide composition of lab-scale bell pepper-PS and carrot-PS hot water extracts.

	Monosaccharides % (mol/mol)								Ratios and Regions				
	Rha	Ara	Gal	Fuc	Glc	Man	Xyl	GalA	GalA/Rha ^1^	RG-I ^2^	RG-I bb ^3^	Ara/Rha ^4^	Gal/Rha ^5^
Bell pepper-PS	2.6	4.0	5.6	0.3	1.9	1.0	1.7	83	32	15	5.2	1.5	2.2
Carrot-PS	2.4	5.6	14.5	0.8	8.7	0.1	0.0	68	28	25	4.8	2.3	6.0

Seaman hydrolysis was used prior to monosaccharide analysis. ^1^ Lower GalA/Rha ratio indicates further enrichment for RG-I backbone, ^2^ RG-I domain calculated as (2*Rha+Ara+Gal), ^3^ RG-I backbone calculated as 2*Rha, ^4^ Ara/Rha and ^5^ Gal/Rha ratio indicate level of neutral Ara and Gal comprising sidechains linked to Rha, respectively. Fuc is fucose.

**Table 4:** Monosaccharide composition of the bpRG-I and cRG-I extracts enriched for RG-I at industrial-scale.

	Monosaccharides % (mol/mol)								Ratios and Regions				
	Rha	Ara	Gal	Fuc	Glc	Man	Xyl	GalA	GalA/Rha ^1^	RG-I ^2^	RG-I bb ^3^	Ara/Rha ^4^	Gal/Rha ^5^
bpRG-I	2.8	6.2	6.9	0.2	28.2	1.2	1.2	53	19	19	5.6	2.2	2.5
cRG-I	9.6	33.9	21.0	0.7	6.1	0.8	0.8	27	3	74	19.2	3.5	2.2
bpRG-I^6^	4.6	7.5	8.0	0.6	29.0	1.9	1.5	47	10	25	9.2	1.6	1.7
cRG-I^6^	14.3	34.8	19.6	0.8	4.3	0.9	0.7	25	2	83	28.6	2.4	1.4

^1^ Lower GalA/Rha ratio indicates further enrichment for RG-I backbone, ^2^ RG-I domain calculated as (2*Rha+Ara+Gal), ^3^ RG-I backbone calculated as 2*Rha, ^4^ Ara/Rha and ^5^ Gal/Rha ratio indicate level of neutral Ara and Gal comprising sidechains linked to Rha, respectively. The first two rows indicate results after Seaman hydrolysis. ^6^ Complementary analyses following methanolysis since Seaman hydrolysis leads to underestimation of Rha [27].

## Supplementary Materials

The following are available online at https://www.mdpi.com/2072-6643/13/3/963/s1, Table S1. Extraction yield and MW distribution of bell pepper-PS and carrot-PS after extraction in hot water (100 °C, 90 min) and bicarbonate (pH 7.5–8, 100 °C, 90 min), Table S2. Secretome in in vitro whole blood assay, Table S3. Impact of bpRG-I and cRG-I on the microbiota composition at the family level (relative abundance) in batch cultures (48 h) with human fecal inoculum, Table S4. Inclusion and exclusion criteria in proof-of-concept human study with bpRG-I, Table S5. Baseline demographic and anthropometric data of the participants.
