# Supplementary material for: Development of an Affordable, Sustainable and Efficacious Plant-Based Immunomodulatory Food Ingredient Based on Bell Pepper or Carrot RG-I Pectic Polysaccharides
Source: Nutrients. 2021 Mar 16;13(3):963. doi: 10.3390/nu13030963 (PMC8002328; doi:10.3390/nu13030963)
Supplement: Supplementary file 1 [file nutrients-13-00963-s001.pdf]

Supplemental material

Running title: The development of an affordable, sustainable and efficacious plant-based immunomodulatory food ingredient based on bell pepper or carrot RG-I pectic polysaccharides

Supplemental table S1. Extraction yield and MW distribution of bell pepper-PS and carrot-PS after extraction in hot water (100°C, 90 min) and bicarbonate (pH 7.5-8, 100°C, 90 min).

material	extraction	Extraction yield (wt%)	Mw distribution >110 kDa (wt%)	70-110 kDa (wt%)	40-70 kDa (wt%)	<40 kDa (wt%)
bell pepper-PS	water	9	12	16	21	51
	$NaHCO_3$	12	18	13	27	42
carrot-PS	water	9	9	16	22	53
	$NaHCO_3$	20	33	23	17	27

MW fractions corresponding to >110 kDa, 70-110 kDa and 40-70 kDa were collected and extraction yields measured. The remainder fraction was considered to have a MW <40 kDa.

Supplemental table S2. Secretome of in vitro whole blood assay.

	Granzyme B (ng/mL)	IFN-gamma (pg/mL)	IL-1 alpha (ng/mL)	IL-1 beta (ng/mL)	IL-12p40 (ng/mL)	IL-15 (ng/mL)	IL-17 (pg/mL)	IL-18 (pg/mL)	IL-7 (pg/mL)	IL-8 (ng/mL)	MCP-1 (ng/mL)	MIP-1 alpha (ng/mL)	MIP-1 beta (ng/mL)	SCF (pg/mL)	TNF-alpha (ng/mL)	TNF-beta (pg/mL)
**pbs**	8	0	0.01	0.02	0.45	0.49	0.4	42	2	0	0	0	0	174	0.0	0
**Apple pectin**	16	1	0.00	0.02	0.48	0.50	1.0	46	28	1	7	0	3	177	0.0	2
**bpRG-I**	21	252	0.04	2.08	4.08	0.79	5.4	92	164	146	6	25	313	450	6.4	76
**cRG-I**	22	148	0.02	1.14	3.51	0.65	3.7	75	159	160	21	23	284	317	3.4	63
**b-glucan**	20	2	0.00	0.01	0.44	0.47	1.4	51	120	18	14	0	13	161	0.0	7
**LPS/SEB**	34	15643	0.04	1.44	4.20	0.65	370	92	103	19	5	25	405	292	5.4	314

	GM-CSF (pg/mL)	IL-10 (pg/mL)	IL-1ra (pg/mL)	IL-4 (pg/mL)	IL-5 (pg/mL)	IL-2 (pg/mL)	IL-23 (ng/mL)	IL-3 (ng/mL)	IL-6 (ng/mL)	MMP-3 (ng/mL)	MMP-9 (ng/mL)	VEGF (pg/mL)	BDNF (ng/mL)	Eotaxin-1 (pg/mL)	Factor VII (ng/mL)	ICAM-1 (ng/mL)	SI
**pbs**	0	0	1333	0	0	5	2.80	0.01	0	3.1	33	74	0.3	287	177	36	
**Apple pectin**	0	4	1907	20	0	5	2.73	0.01	0	3.2	42	80	0.4	298	185	37	>500
**bpRG-I**	25	1999	4750	149	0	82	6.20	0.02	17	3.2	90	239	5.4	315	196	36	>100
**cRG-I**	17	338	3477	162	0	67	5.37	0.02	12	3.2	84	143	3.9	296	181	35	>50
**b-glucan**	0	4	1730	85	0	19	2.34	0.00	0	3.0	50	180	2.8	271	169	32	>10
**LPS/SEB**	842	3147	4700	397	104	13380	5.18	0.06	9	3.3	77	105	2.5	278	179	34	>5

Concentrations per mediator are indicated as average of 3 donors. Color-coded according to stimulation index (SI) vs unstimulated control (PBS) with scale on the right.

Supplemental table S3. Impact of bpRG-I and cRG-I on the microbiota composition at the family level (relative abundance) in batch cultures (48h) with human fecal inoculum.

Phylum	Family	Inoculum	Base medium	bpRG-I	cRG-I
Actinobacteria	Coriobacteriaceae	0.5%	0.5%	2.1%*	0.2%*
Bacteroidetes	Bacteroidaceae	29.3%*	44.7%	54.6%*	69.2%*
	Porphyromonadaceae	0.4%	0.6%	0.9%*	0.2%*
	Rikenellaceae	6.3%*	1.1%	1.1%	0.5%*
Firmicutes	Acidaminococcaceae	0.7%*	9.2%	10.7%	11.2%*
	Erysipelotrichaceae	0.3%*	0.1%	0.4%*	0.1%*
	Lachnospiraceae	33.5%*	18.9%	15.7%*	13.6%*
	Ruminococcaceae	28.6%*	3.7%	6.3%*	2.3%*
Proteobacteria	Alcaligenaceae	0.2%*	2.8%	1.2%*	0.5%*
	Desulfovibrionaceae	0%*	0.4%	0.1%*	0%*
	Enterobacteria	0%*	17.9%	6.9%*	2.2%*

Statistical differences within each family versus the base medium incubation, as calculated with a 2-sided student t-test, are indicated by an asterisk ($p \leq 0.05$). Only families with abundances of at least 0.1% in one of the treatments are shown.

Supplemental table S4. Inclusion and exclusion criteria in proof-of-concept human study with bpRG-I.

Inclusion criteria

- Apparently healthy, Caucasian, males and females, aged 20–65 years at study start
- Body mass index (BMI) of 18.5 to 29.9 kg/m2
- No disorders that may interfere with the study conduct or evaluation, e.g. current or previous respiratory, cardio-vascular or metabolic diseases, chronic (auto)-immune, gastrointestinal disorders, infections etc.
- Values within the normal reference range at screening of leucocytes, lymphocytes and monocytes
- No clinically relevant deviations and/or no deviations > 2 x ULN (upper limit of normal) in other laboratory values at screening
- Readiness to comply with all specific study procedures, in particular:
    - Consumption of the investigational product (IP) during the entire study
    - Maintaining the habitual diet, with the exception regarding the required dietary restrictions throughout the study to be complied with
    - Accepting the collection of fecal samples for evaluation of fecal microbiome
    - Accepting blood draws to evaluate immune response
    - Filling in health questionnaires
- Women of childbearing potential: negative pregnancy testing (ß-HCG (beta subunit of human chorionic gonadotropin) test in urine) at screening and readiness to use reliable contraception methods for the trial duration
- Prerequisite for the participation in the study was a written informed consent by the participant following written and oral information by the investigator regarding nature, purpose, possible benefit and possible risks of the clinical study

Exclusion criteria

- Previously known hypersensitivity to any component of the investigational product and/or to bell pepper
- Known severe acute or chronic immunological disorders such as acquired immune deficiency syndrome (AIDS) (or human immunodeficiency virus (HIV) positive)
- Malignant disease within the last 5 years prior to screening
- Known diagnosed allergies (including any food allergy)
- Untreated or unstable hypertension (>140/90 mmHg)
- Mastopathy and/or menstrual disorders
- Any surgery or vaccination within the last 3 months prior to screening and during the study
- Intake of antibiotics, systemic corticosteroids, immune suppressants, psychotherapeutic treatment that may affect the blood count within the last 3 months prior to screening (except for nonsteroidal anti-inflammatory drugs and other potentially anti-inflammatory local analgesics ≥2 days prior to screening and during study and/or intake of any treatment and/or supplementation influencing the immune system during the study (except for nonsteroidal anti- inflammatory drugs and other potentially anti- inflammatory analgesics ≥2 days prior to study visits)
- Regular use of statins
- Reported, unexplainable weight loss or gain >2 kg in the last month before screening
- Intense sporting activities >10 h/week before screening and during the study
- Participation in night shift work or reported irregular sleep-wake pattern 2 weeks before screening and during the study
- Reported dietary restrictions such as consuming a medically prescribed diet, slimming diet or vegetarian diet in the last 3 months prior to screening or during the study
- Reported total consumption of >10 units (tablets, capsules, sachets etc.) of vitamin supplements containing β-carotene, vitamin C, E or zinc in the last 3 months prior to screening or any such supplementation during the study
- Reported consumption of probiotic foods or drinks in the last month prior to screening or during the study
- Reported alcohol consumption of >2 units/day (1 unit equals approximately 250 mL of beer, 100 mL of wine or 35 mL of spirits)
- Reported history of abuse of drugs or medication in the last 6 months prior to screening
- Regular consumption of tobacco
- Blood donation or transfusion in the last 2 months prior to screening or during the study
- Participation in another study in the last 2 months before screening or during the study
- Participation in the present study of a person living in the same household as the subject

- Plans to travel abroad during the period of the entire study
- Inability to comply with study procedures (e.g. due to language difficulties etc.)
- Pregnancy or lactation
- Any other reason deemed suitable for exclusion, per investigator's judgment

Supplemental table S5. Baseline demographic and anthropometric data of the participants.

Parameter	Total<br><br>n=57	Placebo<br><br>n=20	0.3 g /day<br>(low dose)<br>n=19	1.5 g / day<br>(high dose)<br>n=18
Age	49.0 ± 12.0	48.0 ± 12.9	46.2 ± 13.5	52.5 ± 8.2
Female (#/%)	39/68.4%	14/70.0%	13/68.4%	12/66.7%
Height (cm)	171.5 ± 9.1	171.2 ± 9.3	173.4 ± 9.3	170.0 ± 8.8
Weight (kg)	70.8 ± 11.6	70.2 ± 11.5	70.9 ± 11.4	71.3 ± 12.4
